# Beyond Legal Recognition: Parental Acceptance and Couple-Level Minority Stress on Same-Sex Couples’ Well-Being in Chinese Societies

**DOI:** 10.1007/s10508-026-03539-6

**Published:** 2026-10-06

**Authors:** Eliz Miu Yin Wong, Timothy Hildebrandt, Randolph C. H. Chan, Yiu Tung Suen

**Affiliations:** 1 Department of Social Sciences and Policy Studies, The Education University of Hong Kong, Tai Po, Hong Kong Special Administrative Region China; 2 Department of Social Policy, London School of Economics and Political Science, London, UK; 3 Department of Social Work, United College, The Chinese University of Hong Kong, Shatin, Hong Kong Special Administrative Region China; 4 Gender Studies Programme, The Chinese University of Hong Kong, Shatin, Hong Kong Special Administrative Region China

**Keywords:** Couple-level minority stress, Parental acceptance, Well-being, Same-sex couples, Sexual orientation, Taiwan, Hong Kong

## Abstract

Previous studies in the Global North suggest that same-sex marriage brings positive impacts on the well-being of lesbian, gay, bisexual, queer, and plus (LGBQ+) individuals. Parental acceptance is challenging yet important for LGBQ+ individuals, especially in Chinese societies, which strongly emphasize harmony and heteronormative family relationships. This paper examines the association between the legal recognition of same-sex couple relationships and one’s individual and relational well-being, and how it is mediated by parental acceptance and couple-level minority stress. We surveyed 812 LGBQ+ respondents in committed same-sex relationships in two Chinese-majority societies, Taiwan (with legal recognition of same-sex couple relationships) and Hong Kong (without such policy). We found that Taiwan respondents reported higher levels of parental acceptance and a lower level of couple-level stigma and discrimination than their counterparts in Hong Kong. While Taiwan respondents reported better relational well-being, there was no significant difference in individual well-being between the two societies. Experience of couple-level stigma and discrimination was associated with higher levels of depression. We argue that parental acceptance explains much of the significant variation in relationship satisfaction, beyond living in societies with or without legal recognition of same-sex couple relationships. As such, we underscore the importance of parental acceptance for LGBQ+ individuals in their relationship with their same-sex partners in Chinese societies. Furthermore, as same-sex couples continue to encounter couple-level minority stress and challenges in gaining parental acceptance, there is a need for mental health and social services to provide targeted support to enhance their well-being.

## Introduction

Previous studies in the Global North suggest that same-sex marriage brings positive impacts on the well-being of lesbian, gay, bisexual, queer, and plus (LGBQ+) individuals, as well as same-sex couples (Hatzenbuehler et al., 2017). However, even where marriage equality is implemented, same-sex couples may still experience social stigma and rejection, especially from their families of origin—as well as couple-level minority stress, stigma, and rejection experienced because of their same-sex relationship, beyond their sexual identity (Frost et al., 2017).

In many contexts, such as Chinese societies, the “personal” is also very much “familial” (Hildebrandt, 2019). Parental acceptance remains a major challenge for most of LGBQ+ individuals in Chinese societies. Cultural emphasis on the family makes them seek to integrate their sexualities into their family. Instead of “coming out” (explicitly disclosing their sexualities to their parents), LGBQ+ Chinese often prefer “coming home,” bringing their same-sex partners back home to meet their parents and being accepted gradually into the family (Chow, 2001; Huang & Brouwer, 2018). But how does the political affect the familial? What roles do progressive social policies such as the legal recognition of same-sex couple relationships play in parental acceptance? And, related, how does such legal recognition affect the relational well-being of couples, apart from just individuals?

This paper explores the association between the legal recognition of same-sex couple relationships, parental acceptance, and individual and relational well-being among LGBQ+ individuals in committed same-sex relationships. We conducted a comparative study in two Chinese-majority societies: Taiwan (which enacted the 748 Act—a special law for the legal recognition of same-sex partnerships) and Hong Kong (which lacks such policy). Both are Chinese-majority societies with strong influences of family values (Chan & Huang, 2022; Kong, 2019). LGBQ+ individuals still face widespread discrimination and rejection in these two societies (Huang & Chan, 2022; Suen et al., 2016). A key distinction between these two contexts is the presence of the legal recognition of same-sex couple relationships, which was implemented in Taiwan in 2019 but remains absent in Hong Kong. Drawing on online survey data from 812 respondents in committed same-sex relationships across Taiwan and Hong Kong, this study examines how differences in legal recognition and family dynamics may relate to the well-being of LGBQ+ individuals within these similar cultural contexts.

### Individual-Level and Couple-Level Minority Stress Model

Studies consistently show that the well-being of LGBQ+ individuals is worse than that of heterosexual individuals (Frost et al., 2015; la Roi et al., 2019). A common model to explain this disparity is the minority stress theory (Meyer, 2003), which focuses on the stressors uniquely experienced by LGBQ+ individuals as members of a socially stigmatized group. Against the backdrop of homophobia deeply rooted in historical, cultural, social, and institutional dimensions, LGBQ+ individuals experience a broad range of stigma, concealment, family rejection, discrimination, and bias-based victimization (Fulginiti et al., 2020; Wong et al., 2025). Living in heterosexist environments, LGBQ+ individuals need to constantly evaluate the external environment, maintain vigilance, and conceal their identity to keep themselves safe from rejection and harassment (Bauerband et al., 2019; Chang et al., 2022).

Most studies on the minority stress framework focus on individual-level stressors (LeBlanc et al., 2015; Stewart et al., 2019), which are not well suited to capture the pressures that emerge because of one’s same-sex relationship status. As such, recent studies have developed a relational focus, identifying unique stressors experienced by same-sex couples, which cannot be reduced to their experience as LGBQ+ individuals (Mohr & Daly, 2008; Riggle et al., 2005; Rostosky et al., 2016). The concept of “couple-level minority stress” provides a more holistic understanding of minority stress in the lives of LGBQ+ people not only as an individual but also in their stigmatized same-sex relationship (Frost et al., 2017; LeBlanc et al., 2015).

Couple-level minority stress can be experienced by an individual’s partner, or jointly by the couple, on the grounds of their same-sex relationship (LeBlanc et al., 2015). When one’s same-sex relationship is devalued, stigmatized, or socially marginalized, an individual may face challenges or hardship, which poses negative impacts on their well-being (LeBlanc & Frost, 2020). A quantitative study of 100 dyadic same-sex couples in the USA examined couple-level stigma and its association with psychological distress and risky behaviors, beyond the effect of individual-level stigma (LeBlanc & Frost, 2020). Specifically, the results revealed that stressors targeting the relationship unit—such as the social de-legitimization or lack of recognition of the couple—were associated with higher levels of depressive symptoms and alcohol use, even after controlling for individual-level discrimination (LeBlanc & Frost, 2020). It provides empirical evidence that couple-level minority stressors significantly affect same-sex couples’ well-being beyond the impacts of individual-level minority stressors.

### Individual and Relational Well-Being

Extensive research has demonstrated the association between minority stress and individual-level mental health, encompassing depression (Bruce et al., 2015; Sarno et al., 2020), anxiety (Kelleher, 2009; Puckett et al., 2018), substance use (Lehavot & Simoni, 2011), health-risk behaviors (Hamilton & Mahalik, 2009; Murchison et al., 2017), and suicidality (Fulginiti et al., 2021; Meyer et al., 2021). Moving beyond these individual outcomes, scholars are increasingly underscoring the importance of relational well-being, in addition to individual well-being.

Relational well-being, originating from a relational ontology, views relationships not as external “social support” but as fundamental to one’s well-being (White, 2017). This alternative view challenges the dominant ideology of an autonomous and bounded self by situating one’s well-being within a relational network (Gergen, 2009). Empirical studies adopting this perspective often examine self-reported relationship quality between parents and children, and between couples, to measure one’s satisfaction and experiences in their relationships with others (Flora & Segrin, 2003; Greenfield & Marks, 2006; McDaniel & Drouin, 2019). This perspective is especially crucial when studying marginalized populations, as they often face various relationship challenges such as rejection and isolation due to their marginalized identities, higher self-concealment, and poor relationship quality (Huang & Hang, 2024; Lewis et al., 2021; McCubbin et al., 2013; Rice et al., 2022).

### The Impacts of Parents’ Acceptance on LGBQ+ Individuals’ Well-being

The reaction of parents toward their children’s sexual orientation can serve as a significant risk or protective factor for LGBQ+ individuals’ well-being (Poštuvan et al., 2019). Parental rejection makes LGBQ+ individuals more vulnerable to psychological distress, including depression, anxiety, and suicidality (Feinstein et al., 2014; O’Brien et al., 2016; Puckett et al., 2015). Parental acceptance of children’s sexual orientation, on the other hand, is a strong protective factor against negative health outcomes experienced by LGBQ+ individuals (Goldbach et al., 2014; Padilla et al., 2010; Poštuvan et al., 2019).

Parents’ acceptance or rejection extends beyond their children’s sexual orientation to include their children’s same-sex relationships. Romantic relationships, including mixed-sex or same-sex partnerships, are deeply embedded in social networks, where network approval bolsters positive relationship outcomes and disapproval is associated with relationship termination (Sprecher & Felmlee, 1992). Perceived support from family and friends is positively associated with relationship quality, enhancing feelings of love, satisfaction, and commitment (Sinclair et al., 2015; Sprecher & Felmlee, 1992). Furthermore, social support specifically directed at a romantic relationship is an empirical construct distinct from general social support; it acts as a unique predictor of relationship well-being, which subsequently influences both mental and physical health (Blair et al., 2018). However, these network dynamics operate differently for marginalized couples, who perceive significantly more social disapproval and marginalization than non-marginalization couples (Lehmiller & Agnew, 2006). For individuals in same-sex relationships, perceived support from family members is often significantly lower than that reported by individuals in mixed-sex relationships (Holmberg & Blair, 2016). Moreover, in same-sex relationships, support for a specific relationship is often deeply intertwined with support for an individual’s broader sexual orientation, reflecting the concept of attributional ambiguity (Blair & Holmberg, 2019; Holmberg & Blair, 2016). Whereas heterosexual individuals take affirmation of their sexual identity for granted, LGBQ+ individuals may interpret a lack of support as reflecting broader homonegativity or prejudice toward same-sex relationships, rather than dislike of a particular partner (Blair & Holmberg, 2019; Holmberg & Blair, 2016).

Couple-level minority stress is most frequently and acutely experienced by same-sex couples within familial contexts (Frost et al., 2017). Same-sex couples face challenges to participate in their families of origin, for example, they are not welcome to join family gatherings or spend time with their relatives’ children as a couple. These challenges may be based on one’s same-sex relationship, rather than just their own sexualities. For example, a man who is gay may be able to join his family’s event on his own, but he may not be welcomed if he brings his same-sex partner (Frost et al., 2017). Rejection experienced by same-sex couples in their or their partner’s families of origin may lead to the couple’s conflicts, especially when partners have diverse views on how to navigate the family’s rejection (Hoff et al., 2024). Also, same-sex couples may experience a lack of social support from their families when they face relationship problems due to the devaluation of their same-sex relationship by their families (Frost et al., 2017).

However, same-sex couples are not merely passive recipients of familial pressures; they can exercise agency by proactively navigating parental opposition. Research on support marshaling behaviors conceptualizes these practices as strategic communicative efforts to solicit support and to buffer the relationship from family interference (Crowley, 2012). Such strategies are categorized along two dimensions: approach versus avoidance (seeking to enhance support versus evading further opposition) and direct versus indirect (explicitly addressing network members versus keeping efforts covert). By deploying these actions, couples may manage parental disapproval in ways that protect dyadic well-being and foster a more resilient relational climate amid marginalization (Crowley, 2012).

### The Impacts of Legal Recognition of Same-sex Couple Relationships on LGBQ+ Individuals’ Well-being

Extant literature demonstrates that unequal legal recognition of same-sex relationships plays a vital role in minority stress experienced by same-sex couples (Boertien & Vignoli, 2019; Cao et al., 2017; Hatzenbuehler et al., 2017; Ogolsky et al., 2019a, 2019b). Same-sex couples face challenges regarding the legitimacy of same-sex relationships, which come from both internal self-doubts and external skepticism expressed by their families and friends (H. Liu et al., 2020). Same-sex couples also need to put extra emotional and material costs and effort into mitigating the potential harms of not having legal recognition of their same-sex relationships (e.g., making a will). Moreover, they face difficulties in anticipating the future with their same-sex partners because of the legal precarity (Alston-Stepnitz et al., 2020).

In the USA, prior to the federal legalization of same-sex marriage, LGBQ+ individuals living in states with equal marriage rights reported lower psychological distress and better self-assessed health than those who lived in states without marriage equality (Drabble et al., 2022; Hatzenbuehler et al., 2017; Kail et al., 2015; Raifman et al., 2017). A longitudinal study examined the well-being of LGBQ+ individuals before and after the US Supreme Court’s *Obergefell v. Hodges* [2015] case decision (legalizing same-sex marriage across the nation). After the ruling, the level of stigma experienced by LGBQ+ individuals, particularly those in a same-sex relationship, dropped. Additionally, the level of family support increased following the ruling. This was likely because the Supreme Court ruling made family members feel more comfortable supporting their LGBQ+ relatives as they perceived it as more socially acceptable to do so (Ogolsky et al., 2019b).

Additionally, legal recognition may extend beyond the mitigation of stress, altering the broader social and environmental cues available to LGBQ+ individuals. This aligns with social safety theory (Diamond & Alley, 2022), which posits that the health consequences of stigma derive not only from the presence of stressors but also from the profound absence of social safety—defined as the reliable experience of social connection, inclusion, and protection. From this perspective, humans are biologically prepared to monitor social threat, and sustained downregulation of this vigilance depends on the presence of reliable safety cues. Affirmative laws and policies, such as same-sex marriage, serve as macro-level signals of safety that foster the physiological and psychological health of sexual minority populations by creating a climate of safety at the community level (Diamond & Alley, 2022).

To date, 38 jurisdictions have legalized same-sex marriage (Human Rights Campaign, 2025). Research in “post-marriage equality era” (Hull, 2018) contexts has examined how access to marriage institutions impacts the lived experiences of same-sex couples and LGBQ+ individuals in general (Frost et al., 2022; Kerrigan, 2021; Masri, 2018). A US study examined the possible impacts of recent social and policy changes, including same-sex marriage and the changes in public opinions toward LGBQ+ individuals, on diminishing the negative impacts of minority stress and improving same-sex relationship quality (Frost et al., 2022). It found that emerging adults’ (aged 18–25) same-sex relationship quality is less affected by the proximal forms of minority stress (felt stigma, stigma concealment, and internalized stigma) than that of their older cohort. However, everyday experiences of discrimination are still associated with poorer relationship quality across all cohorts.

That said, even after same-sex marriage is legalized, it does not mean that same-sex couples no longer experience social stigma and minority stress. As Meyer (2016) cautiously notes, “It is important to remember that social equality is not synonymous with equality under the law” (p. 1358). Social stigma and discrimination may persist, even after the discriminatory marriage policy is lifted. More empirical studies are needed to have a thorough understanding of the changes in minority stress at both the individual and couple level, and its impacts on their individual and relational well-being in the post-marriage equality era.

### LGBQ+ Individuals and Parental Acceptance in Chinese Societies

Living in Confucian-dominant cultures, which value family responsibilities and collectivism, LGBQ+ individuals in Chinese societies face tremendous pressure from their parents to fulfill their filial obligation to get (heterosexually) married and have children to continue the family lines. As such, being gay is perceived as not only a personal issue, but also a family “problem” (Chow, 2001; Hildebrandt, 2019). LGBQ+ individuals may bring shame to their families, causing parents to “lose face” in their communities (Choi & Luo, 2016; Kwok & Wu, 2015). A recent study with over 10,000 lesbian and gay adults in Mainland China revealed that the vast majority (70.4%) face family pressure to get (heterosexually) married and have children, and more than half of them (54.2%) have experienced negative treatment and violence related to their sexual orientation in their families of origin (Suen et al., 2023a, 2023b). Studies in Hong Kong and Taiwan also demonstrated how LGBQ+ individuals face tremendous difficulties in coming out to their parents and getting their parents’ acceptance (Brainer, 2018; Huang et al., 2020; Suen et al., 2023a, 2023b).

Studies show that LGBQ+ individuals may adopt different strategies in coming out and gaining acceptance from their parents. The model of “coming home,” first developed by Chow (2001), distinguishes the difference in sexual politics and coming out approaches in the West and in societies under the influence of Chinese culture. Chow (2001) argues that rather than coming out explicitly (disclosing one’s sexual orientation to their parents), LGBQ+ individuals in Chinese societies seek to first integrate their sexuality into their family/kin network. Family relationships and harmony are perceived as vital, if not more so, than one’s sexualities (Chan, 2017; Chow, 2001). LGBQ+ individuals in these societies often choose to bring their same-sex partner home and introduce their partner to their parents without explicitly discussing their sexualities. Then, their partner is gradually accepted by their parents as their “second son/daughter” and becomes part of the family (Huang & Brouwer, 2018). “Coming home” has been widely discussed in LGBQ+ studies in a Chinese context (Engebretsen, 2013; Liu & Ding, 2005; Wei & Yan, 2021). Wong (2007) found that for LGBQ+ individuals in Hong Kong, the coming home approach is commonly adopted and it is perceived as indispensable to coming out. Studies in Mainland China explored the efforts LGBQ+ individuals put into trying to integrate their same-sex relationships into their families of origin and get their parents’ acceptance (Huang & Brouwer, 2018; Wei & Yan, 2021). Jhang (2018) found that Taiwanese LGBQ+ individuals choose to bring their same-sex partner home to meet their parents, but not explicitly come out to their parents in order to maintain a stable family relationship. This “coming home” model highlights how LGBQ+ individuals in Chinese societies intend to integrate their sexualities and same-sex relationships into familial connections, rather than pursue non-traditional and individualized family lives (Chan, 2017).

In 2019, the Taiwan Legislature enacted the Act for Implementation of J.Y. Interpretation No. 748 (the “748 Act”), a special law granting legal recognition to same-sex unions. Notably, the 748 Act was a compromise “special law” that deliberately avoided using the word “marriage,” even as it conferred many of the rights associated with marriage under the Civil Code (Friedman & Chen, 2023). Rather than amending the Civil Code to include same-sex relationships, same-sex couples register their union under this separate special law. While the legislation is formally titled the “748 Act,” the term “marriage” for same-sex couples is used across official government documents and websites, including the marriage registration forms utilized by same-sex couples (www.gov.tw, 2024). Acknowledging this distinction is vital, as the use of a special law rather than the Civil Code reflects ongoing societal compromises, marginalization, and the unique legal positioning of LGBTQ + rights in Taiwan. To avoid confusion and improve clarity, we use the term “legal recognition of same-sex couple relationships” throughout the manuscript to refer to the “748 Act.”

This change in legal recognition of same-sex couple relationships provide an excellent opportunity to examine how such policy affects LGBQ+ individuals and their interaction with their parents in predominantly Chinese society. Huang and Hang’s (2024) qualitative study of 15 LGB adults in Taiwan explores the effects of legal recognition on their relationships with parents and partners. While the legislation did not drastically change parental attitudes, LGB respondents felt it had a positive influence on their relationships, fostering stronger bonds and open dialogue with their parents. Regarding romantic relationships, the study finds no immediate changes in relationship quality or a sudden increase in engagement in relationships. However, participants felt the legislation gave them a sense of hope and laid a foundation for developing intimate relationships. These findings shed light on the influence of legal recognition on the relationships of LGBQ+ individuals with their parents and partners. Further research is needed to fully understand the multidimensional impacts of this policy on the well-being of LGBQ+ individuals.

### Research Gaps

This paper seeks to fill three significant gaps in the literature. First, existing studies primarily focus on minority stress and its impacts on well-being on an individual level. They may not be able to capture the unique stressors faced by LGBQ+ individuals on the grounds of their same-sex relationship and its impacts on their relational well-being (Frost et al., 2017; LeBlanc et al., 2015). Second, most studies on parental rejection and acceptance focus on LGBQ+ youth (Poštuvan et al., 2019). While romantic relationships are deeply embedded in social networks and influenced by network approval (Blair & Holmberg, 2019; Sprecher & Felmlee, 1992), research on how LGBQ+ adults navigate these dynamics with their families of origin remains limited (Hoff et al., 2024; Reczek, 2020). The relative lack of research concerning LGBQ+ adults and their relationships with parents may be partly attributed to the assumption that the significance of parental attitude toward their children’s sexual orientation diminishes as their children reach adulthood. But this assumption is not necessarily valid across cultures (Acosta, 2013). For example, lesbian and gay adults in China have experienced violence based on their sexual orientation from their families of origin across different age-groups. Such a study highlights that the negotiation between parents and children on their sexual orientation persists into adulthood (Suen et al., 2023a, 2023b). Third, studies on the legal recognition of same-sex couple relationships mainly focus on North American and European countries, with the highlight of individualism and personal choice (Umberson et al., 2018). They may not be able to capture the complexity of culture. In particular, under the influence of Chinese culture, which emphasizes family relationships and harmony, the relationship among legal recognition, family acceptance, couple-level minority stress, and individual and relational well-being of LGBQ+ individuals in committed same-sex relationships should be examined thoroughly.

### The Current Study

This study fills these research gaps by conducting a comparative study on LGBQ+ individuals in a committed same-sex relationship who live in Taiwan (with the legal recognition of same-sex couple relationships) and Hong Kong (without such policy). This study aims to understand the difference in well-being of LGBQ+ individuals in committed same-sex relationships who live in societies with and without legal recognition, and how the well-being disparity can be explained by the policy difference, parental acceptance, and couple-level minority stress.

The two cases compared in this paper, Hong Kong and Taiwan, are Chinese-majority societies with similar cultural values that emphasize family structure and are deeply rooted in Confucianism (Kong, 2019). Previous studies reveal that LGBQ+ individuals in both regions are marginalized and face widespread discrimination and rejection (Huang et al., 2025; Suen et al., 2016; Wong et al., 2025). The main difference, however, is the legal recognition of same-sex couple relationships. In 2019, Taiwan became the first and only region in Asia which enacted legislation for same-sex partnerships, while there is no such policy in Hong Kong.

This study aims to (1) identify the difference in parental acceptance, couple-level stress and discrimination, and individual and relational well-being among LGBQ+ individuals who live in Taiwan (a society with legal recognition of same-sex couple relationships) and Hong Kong (a society without this policy), (2) investigate the association between one’s residence in societies with or without legal recognition, parental acceptance, couple-level stress and discrimination, and individual and relational well-being, (3) determine the mediating role of parental acceptance and couple-level minority stress on the association between one’s residence and their individual and relational well-being.

Given that the previous literature demonstrates the positive impacts of legal recognition on LGBQ+ well-being and their relationship with their parents, we propose the following hypotheses: LGBQ+ individuals in committed same-sex relationships who live in Taiwan would report higher levels of parental acceptance of sexual orientation (Hypothesis 1) than their counterparts in Hong Kong. Also, respondents in Taiwan would report lower levels of couple-level stress and discrimination (Hypothesis 2). Moreover, we hypothesized that respondents in Taiwan would have better individual and relational well-being, which is lower levels of depression and higher levels of relationship satisfaction (Hypothesis 3).

In addition, we propose two hypothesized models, in which parental acceptance and couple-level stress and discrimination would mediate the association between one’s residency and their individual well-being (i.e., depression, model 1) and relational well-being (i.e., relationship satisfaction, model 2), respectively.

## Method

### Participants and Procedure

This paper is part of a larger collaborative research study on LGBQ+ individuals living in Hong Kong and Taiwan conducted from May 2021 to March 2022. Inclusion criteria for the larger study were individuals who (1) were aged 16 or above; (2) self-identified as gay, lesbian, bisexual, or otherwise non-heterosexual; (3) were living in Hong Kong or Taiwan; and (4) were able to understand Chinese. Participants were recruited through local LGBQ+ community organizations and social media in Hong Kong and Taiwan. Participants who showed an interest were invited to read the study background and give their informed consent before they started answering the online survey hosted by Qualtrics. A gift voucher of HK$50 (around US$6) or NT$150 (around US$5) was offered to each participant as compensation for their time. The data were anonymized to protect the confidentiality of the participants. 

Given that this study focuses on the legal recognition of same-sex couple relationships, only respondents who were in a committed relationship with their same-sex partner, rather than those who were single, are included because such policy is the most proximate to same-sex committed couples’ situation. The legal age of forming such union is 18 years old in Taiwan, so only respondents who were aged 18 or above are included. The unions of transgender and gender non-binary individuals usually involve other laws and policies, including legal gender recognition (Dunne, 2014). In order to focus on the policy of interest in this paper, legal recognition of same-sex couple relationships, this study only includes cisgender individuals in the analysis. Moreover, this study examines the impacts of parental acceptance on LGBQ+ individuals’ well-being and only respondents who had contact with at least one of their parents and whose parents are not same-sex couples (rainbow family) are included. Also, respondents who chose “refused to answer” to all 10 items in the Couple-Level Minority Stress (CLMS) scale were excluded from the analysis.

To ensure the validity and integrity of the survey responses, several rigorous screening procedures were implemented. First, the dataset was screened for duplicate Internet Protocol addresses to prevent multiple entries from the same source. Second, to assess respondent attentiveness, two validity items (attention checks) were strategically embedded within the questionnaire; individuals who failed to provide the correct response to these items were excluded from the analytical sample. Finally, a time-completion audit was conducted. Based on pretesting, which indicated an average completion time of approximately 30 min, a threshold was established to exclude respondents who completed the survey in less than 15 min. Such rapid completion often signifies speeding and potentially indicates unreliable data.

A total of 812 respondents in committed same-sex relationships from Taiwan (*n* = 414, 51.0%) and Hong Kong (*n* = 398, 49.0%) constituted the final analytical sample. More respondents self-identified as female (56.4%) than male (43.6%). The mean age was 29.31 (S.D. = 6.82). Nearly a tenth of the respondents (9.0%) were married to their same-sex partners. The majority had an undergraduate degree or a higher level of education (88.7%). More than half (65.1%) earned less than the average monthly income in their respective regions. Table 1 describes the demographics of the total sample as a whole and by region.

**Table 1:** Demographics of the respondents

	Analyzed sample (*n* = 812) N (%)/Mean (S.D.)	Taiwan (*n* = 414) N (%)/Mean (S.D.)	Hong Kong (*n* = 398) N (%)/Mean (S.D.)	Group difference χ^2^/*t* value
*Demographics*				
Gender				
Men	354 (43.6%)	190 (45.9%)	164 (41.2%)	1.81
Women	458 (56.4%)	224 (54.1%)	234 (58.8%)	
Age				
18–29	456 (56.2%)	218 (52.7%)	238 (59.8%)	4.20*
30 or above	356 (43.8%)	196 (47.3%)	160 (40.2%)	
Education				
Below undergraduate	92 (11.3%)	37 (8.9%)	55 (13.8%)	4.81*
Undergraduate or above	720 (88.7%)	377 (91.1%)	343 (86.2%)	
Monthly income				
Below average regional income	529 (65.1%)	340 (82.1%)	189 (47.5%)	107.23***
On or above average regional income	283 (34.9%)	74 (17.9%)	209 (52.5%)	
Marital status				
Committed relationship	739 (91.0%)	364 (87.9%)	374 (94.2%)	9.84**
Married	73 (9.0%)	50 (12.1%)	23 (5.8%)	
Parental acceptance				
Mother’s acceptance	350 (44.8%)	198 (50.0%)	152 (39.4%)	8.92**
Father’s acceptance	231 (33.3%)	128 (36.5%)	103 (30.1%)	3.14
Parental acceptance (1–5)	3.11 (1.17)	3.23 (1.15)	2.98 (1.18)	− 3.02**
Couple-level minority stress				
Stigma (0–4)	1.33 (0.83)	1.17 (0.82)	1.50 (0.82)	5.63***
Discrimination (0–4)	0.24 (0.43)	0.20 (0.40)	0.28 (0.46)	2.66**
Well-being				
Depressive symptoms (0–27)	6.07 (5.59)	6.17 (5.77)	5.96 (5.40)	− 0.54
Relationship satisfaction (1–21)	15.69 (3.60)	16.10 (3.66)	15.26 (3.49)	− 3.33 ***

*p < .05, **p < .01, ***p < .001

### Measures

*Demographics*. Participants were asked to report their sex assigned at birth, self-identified gender identity, age, educational level, monthly income, marital status, and city of residence. In this study, only cisgender LGBQ+ individuals (i.e., their sex assigned at birth was consistent with their self-identified gender) were included.

*Parental acceptance*. Participants were asked to assess their parental acceptance of their sexual orientation by answering the question “To what extent does your mother/father accept your sexual orientation?” on a 5-point Likert scale from 1 (Not accepting at all) to 5 (Strongly accepting). The option “I have no contact with my mother/father at all or my mother/father passed away” was also given. A mean was calculated from their responses of their mother’s and father’s acceptance (or from either mother’s or father’s if they had contact with only one parent or one parent had passed away) into the variable, parental acceptance. Respondents who indicated they had no contact with both of their parents or both parents had passed away were not included in this study.

While we acknowledge that parental acceptance of an LGBQ+ identity is conceptually distinct from support for a specific same-sex relationship, this study focuses on the former for two primary reasons. First, in the context of same-sex relationships, these constructs are often functionally intertwined due to “attributional ambiguity” (Blair & Holmberg, 2019; Holmberg & Blair, 2016). Unlike heterosexual individuals, who may perceive a lack of support as a critique of a specific partner’s traits, LGBQ+ individuals often interpret familial disapproval of a relationship as a reflection of broader homonegativity or prejudice toward their underlying sexual orientation. Accordingly, assessing parental attitudes toward an individual’s sexual orientation offers a clear lens through which broader family attitudes are often experienced and interpreted by LGBQ+ individuals. Second, the specific cultural nuances of the “coming home” model in Chinese societies complicate the explicit measurement of relationship-specific support (Chow, 2001). As same-sex couples often introduce partners to their families of origin under the guise of “close friends” or “roommates” to maintain familial harmony, the relationship itself is frequently not explicitly named or acknowledged as a romantic union (Huang & Brouwer, 2018; Jhang, 2018). This strategic ambiguity makes it empirically difficult for respondents to assess parental support for a “relationship” that has not been formally disclosed to the parents. As such, assessing acceptance of sexual orientation may serve as a pragmatic proxy for capturing the general perceived sense of inclusion from their parents.

*Couple-level minority stress (stigma and discrimination)*. To assess how much respondents’ worries are attributable to their same-sex relationship (e.g., “Strangers will harm us if we display affection in public”), we used a 10-item Couple-Level Stigma subscale from the CLMS (Neilands et al., 2020). Items were rated on a 5-point Likert scale from 0 (Not at all) to 4 (A great deal), with higher scores indicating greater levels of couple-level stigma. The option “Decline to answer” was provided. A mean score was calculated from the responses in this subscale. Cronbach’s alpha of the scale in this study was 0.84.

To assess how frequently the respondents experienced discrimination based on their same-sex relationship in the past year (e.g., “People we know asked that we not show affection toward one another in their presence”), we used a 10-item couple-level discrimination subscale from the CLMS (Neilands et al., 2020). Items were rated on a 5-point Likert scale from 0 (Never) to 4 (Always). Higher scores indicated greater levels of couple-level discrimination. The option “Decline to answer” was provided. A mean score was calculated from the responses in this subscale. Cronbach’s alpha of the scale in this study was 0.90.

*Individual well-being: Depression*. For individual well-being, a nine-item Patient Health Questionnaire (PHQ-9) (Kroenke et al., 2001) was adopted to measure the severity of depressive symptoms of the respondents over the previous two weeks. Items were rated on a 4-point Likert scale from 0 (Not at all) to 3 (Nearly every day). Cronbach’s alpha of the scale in this study was 0.90. A cutoff score of 10 or above on the total score indicates probable clinical depression. When using the cutoff score of 10, the PHQ-9 has a sensitivity of 88% and a specificity of 88% for the diagnosis of major depressive disorder (Kroenke et al., 2001).

*Relational well-being: relationship satisfaction*. A four-item Couples Satisfaction Index (CSI-4) (Funk & Rogge, 2007) was used to measure the degree of satisfaction they feel for their relationship (e.g., “Having a warm and comfortable relationship”). Items were rated on a 6-point Likert scale from 0 to 5, except for the first item, which was rated on a 7-point Likert scale from 0 to 6. The scale has a score ranging from 0 to 21. A cutoff score of 13.5 or below indicates having a distressed relationship, while a score above 13.5 indicates having a satisfied relationship (Funk & Rogge, 2007). Cronbach’s alpha of the scale in this study was 0.91.

### Data Analysis

Descriptive statistics were used to characterize the demographics, parental acceptance, and well-being (i.e., depression and relationship satisfaction) for the analyzed sample as a whole and by region (i.e., policy difference). Chi-square tests and independent-sample t tests were used to examine the regional difference in demographics, parental acceptance, couple-level stigma and discrimination, and individual and relational well-being.

Pearson’s correlation coefficients were used to examine the bivariate relationship between the study variables. Hierarchical multiple regression analyses were conducted to (1) identify the effect of regional difference on parental acceptance and couple-level stress and discrimination and (2) examine the effect of regional difference, parental acceptance, and couple-level stress and discrimination on one’s individual and relational well-being, adjusting for demographic variables (i.e., gender, age, monthly income level, educational level, and marital status). All demographic variables were dummy coded for the analysis and put in Block 1. The regional variable was put in Block 2. We then included parental acceptance and couple-level minority stress (i.e., stigma and discrimination) as the predictors of well-being in the third and fourth blocks of the analysis. The analyses were conducted using SPSS version 25.0.

We conducted path analysis to test whether parental acceptance, couple-level stigma, and couple-level discrimination would mediate the association of regional difference and individual and relational well-being (i.e., depression and relationship satisfaction), after controlling for demographics. By using PROCESS macro 4.1 for SPSS (Hayes, 2022), we conducted mediation analyses to investigate the mediating role of parental acceptance and couple-level stress and discrimination in the relationship between one’s residency and their individual well-being (Model 1) and relational well-being (Model 2), controlling for demographic variables. The analysis was conducted using 5000 bias-corrected bootstrap samples to generate 95% bias-corrected confidence interval (CI), in order to determine the significance of the conditional indirect effects. The indirect effects were significant if the 96% CI did not include zero.

## Results

### Differences between Respondents Who Lived in Taiwan and Hong Kong

Less than half of all respondents (44.8%) reported that their mother had accepting attitudes toward their sexualities; only one-third (33.3%) reported that their father held accepting attitudes. But there was significant variation across the two study sites. Taiwan respondents were more likely to report their mothers accepted their sexualities, compared to those who lived in Hong Kong (50.0% vs. 39.4%, *χ*^2^ = 8.92, *p* = .003). An independent-samples t test indicated that respondents in Taiwan reported a higher degree of acceptance from their parents (*t* =  − 3.02, *p* = .003), compared to those in Hong Kong. Moreover, respondents in Taiwan experienced a lower degree of couple-level stigma (*t* = 5.63, *p* < .001) and couple-level discrimination (*t* = 2.66, *p* = .004) than their counterparts in Hong Kong.

Nearly one-fourth of all respondents met the criteria for probable clinical depression (22.7%). There is no significant difference in depression among respondents who lived in Taiwan (M = 6.17, SD = 5.77) and Hong Kong (M = 5.96, SD = 5.40); *t*(810) =  − 0.54, *p* = 0.59.

A vast majority of respondents (70.9%) felt satisfied in their committed relationship with their same-sex partners. Taiwan respondents (M = 16.1, SD = 3.66) were more likely to report a satisfying relationship, compared with those who lived in Hong Kong (M = 15.26, SD = 3.49); *t*(810) =  − 3.33, *p* < .001.

Correlation analyses showed that parental acceptance was negatively related to couple-level stigma, discrimination, and depression. It was also positively related to relationship satisfaction. Couple-level stigma and discrimination were positively related to depression and negatively related to relationship satisfaction. Table 2 shows the intercorrelation of parental acceptance, couple-level minority stress, and well-being variables.

**Table 2:** Descriptive statistics and intercorrelations of the variables

	1	2	3	4	5
1 Parental acceptance	–				
2 Couple-level stigma	− 0.91**	–			
3 Couple-level discrimination	-0.09*	0.40**	–		
4 Depressive symptoms	-0.08*	0.24**	0.18**	–	
5 Relationship satisfaction	0.17**	− 0.14**	− 0.18**	− 0.20**	–
Range	1–5	0–4	0–4	0–27	1–21
Mean	3.11	1.33	0.24	6.11	15.67
SD	1.17	0.83	0.43	5.63	3.60
α	0.82	0.84	0.90	0.90	0.91

*p < .05, **p < .01, ***p < .001

### Regional Difference in Parental Acceptance and Couple-level Minority Stress

After adjusting for demographics, respondents in Taiwan were likely to have more accepting parents (*β* = 0.11, *p* = .003) than those who lived in Hong Kong. Also, they were more likely to report lower levels of couple-level stigma (*β* =  − 0.19, *p* < .001) and discrimination (*β* =  − 0.09, *p* = .04) (see Table 3). The results support Hypotheses 1 and 2.

**Table 3:** Effect of demographics and region on parental acceptance and couple-level stigma and discrimination

	Parental acceptance		Couple-level stigma		Couple-level discrimination	
	*B* (SE)	*β*	B (SE)	*β*	B (SE)	*β*
*Block 1: Demographics*						
Gender						
Male (reference category)	–	–	–	–	–	–
Female	0.18 (0.08)	0.08*	-0.03 (0.06)	-0.02	− 0.04 (0.03)	− 0.05
Age						
18–29 (reference category)	–	–	–	–	–	–
30 or above	0.24 (0.09)	0.10**	− 0.08 (0.06)	− 0.05	0.04 (0.03)	0.04
Education						
Below undergraduate (reference category)	–	–	–	–	–	–
Undergraduate or above	− 0.29 (0.13)	− 0.08*	0.08 (0.09)	0.03	− 0.11 (0.05)	− 0.08*
Monthly income						
Below average national income (reference category)	–	–	–	–	–	–
On or above average national income	0.19 (0.10)	0.08*	− 0.05 (0.07)	-0.03	0.02 (0.04)	0.02
Marital status						
Committed relationship (reference category)	–	–	–	–	–	–
Married	1.00 (0.14)	0.24***	− 0.40 (0.10)	-0.14***	− 0.04 (0.06)	− 0.03
R^2^	0.11		0.04		0.02	
F	19.29***		5.98***		2.50*	
*Block 2: Region*						
Hong Kong (without the policy) (reference category)	–	–	–	–	–	–
Taiwan (with the policy)	0.26 (0.09)	0.11**	− 0.32 (0.06)	− 0.19***	− 0.07 (0.03)	− 0.08*
R^2^	0.12		0.07		0.02	
R^2^ change	0.01		0.03		0.01	
F change	8.78**		24.65***		4.3*	

*p < .05, **p < .01, ***p < .001

### Association of Regional Difference, Parental Acceptance, and Couple-level Minority Stress with Well-Being

Table 4 shows the results of regression models testing the associations between regional difference, parental acceptance, couple-level minority stress, and well-being.

**Table 4:** Effect of demographics, region, parental’ acceptance, and couple-level stigma and discrimination on depression and relationship satisfaction

	Depression			Relationship satisfaction	
	*B* (SE)	*β*		*B* (SE)	*β*
*Block 1: Demographics*					
Gender					
Male (reference category)	–	–		–	–
Female	0.83 (0.38)	0.07***		0.72 (0.25)	0.10**
Age					
18–29 (reference category)	–	–		–	–
30 or above	− 0.50 (0.42)	− 0.04		− 0.56 (0.27)	− 0.08*
Education					
Below undergraduate (reference category)	–	–		–	–
Undergraduate or above	− 0.94 (0.61)	− 0.05		0.13 (0.40)	0.01
Monthly income					
Below average national income (reference category)	–	–		–	–
On or above average national income	− 1.75 (0.46)	− 0.15***		0.78 (0.30)	0.10**
Marital status					
Committed relationship (reference category)	–	–		–	–
Married	0.61 (0.7)	0.03		1.06 (0.46)	0.08*
R^2^	0.04			0.03	
F	6.35***			5.33***	
*Block 2: Region*					
Hong Kong (without the policy) (reference category)	–	–		–	–
Taiwan (with the policy)	0.25 (0.42)	0.02		0.89 (0.28)	0.12***
R^2^	0.04			0.05	
R^2^ change	0.00			0.02	
F change	0.43			15.79***	
*Block 3: Parental acceptance*					
Parental acceptance (1–5)	− 0.05 (0.18)	− 0.01		0.35 (0.11)	0.11**
R^2^	0.04			0.07	
R^2^ change	0.00			0.02	
F change	3.62			13.15***	
*Block 4: Couple-level minority stress*					
Stigma (0–4)	1.34 (0.26)	0.20***		− 0.10 (0.17)	-0.02
Discrimination (0–4)	1.51 (0.48)	0.12**		− 1.26 (0.31)	-0.15***
R^2^	0.11			0.09	
R^2^ change	0.07			0.03	
F change	29.12***			11.00***	

*p < .05, **p < .01, ***p < .001

After adjusting for demographics, respondents who were in Taiwan are likely to report higher levels of relationship satisfaction (*β* = 0.12, *p* = .001), compared to those who lived in Hong Kong. However, their depression levels did not significantly differ from those in Hong Kong (*β* = 0.02, *p* = .55). This result partly supports Hypothesis 3.

After controlling for demographics and one’s residency, the respondents whose parents were more accepting are more likely to report higher levels of relationship satisfaction (*β* = 0.11, *p* = .002), compared with those whose parents were less accepting. However, their depression levels do not significantly differ from those whose parents were less accepting (*β* = -0.01, *p* = .79).

In addition, after controlling for demographics, the societies they lived in, and parental acceptance, couple-level discrimination was negatively related to relationship satisfaction (*β* = − 0.15, *p* < .001). Both couple-level stigma (*β* = 0.20, *p* < .001) and discrimination (*β* = 0.12, *p* = .002) were positively related to depression.

### Hypothesized Models of Regional Difference on One’s Well-Being

We tested whether parental acceptance, couple-level stigma, and couple-level discrimination would mediate the association of regional difference with individual well-being (measured as depression, Model 1) and relational well-being (measured as relationship satisfaction, Model 2), after controlling for demographic covariates.

**Fig. 1:**
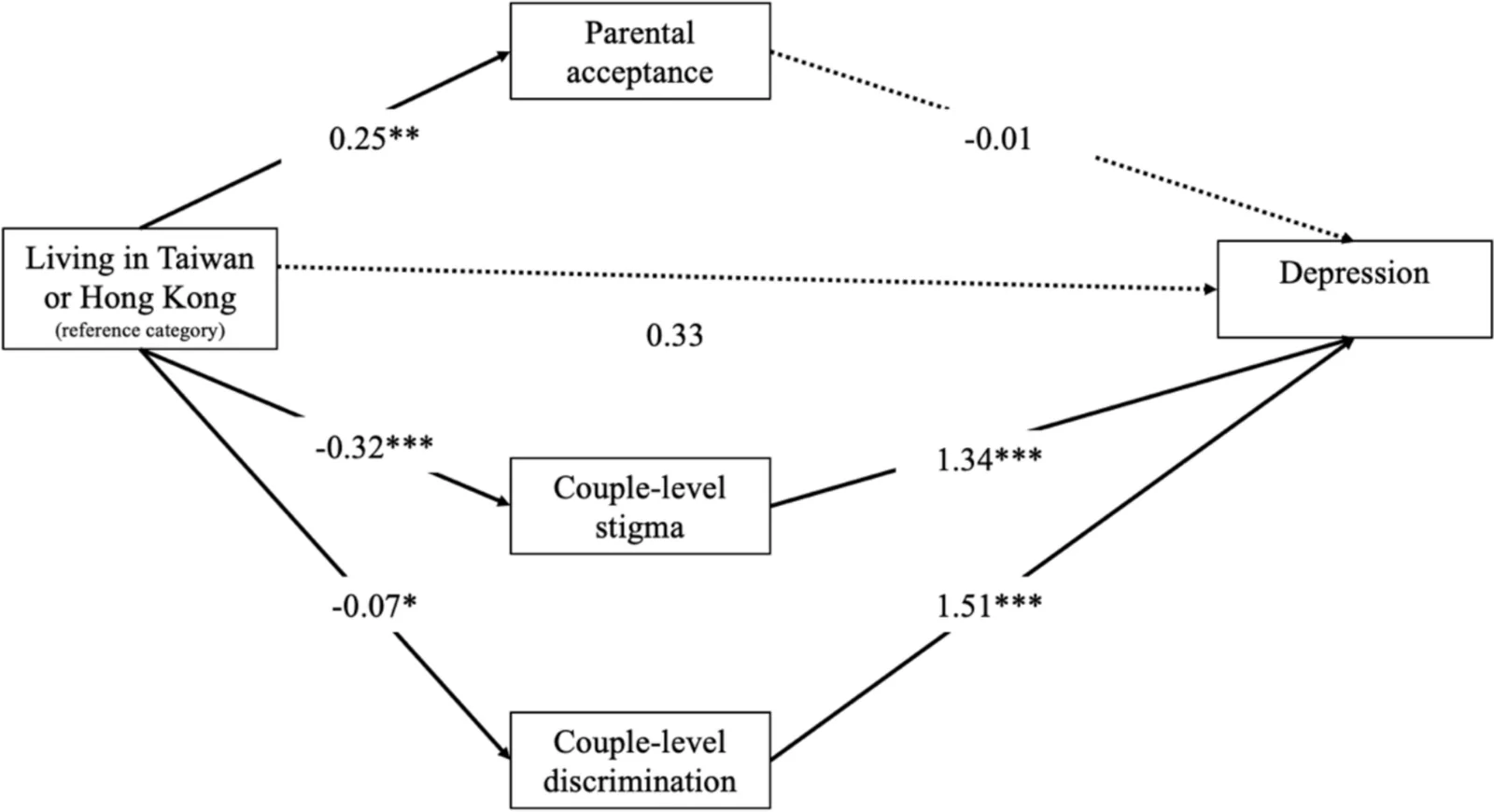
The mediating effect of parental acceptance and couple-level stigma and discrimination in the relationship between the societies one lived in and their depression level. Solid lines indicate significant paths, and dashed lines indicate nonsignificant paths. Demographic variables (gender, age, education, monthly income, and marital status) were controlled for in the analysis. *p < .05, **p < .01, ***p < .001

In Model 1, there are significant indirect effects between the societies one lived in and their depression level through couple-level stigma (*B* =  − 0.42, 95% CI [ − 0.66,  − 0.22]) and couple-level discrimination (B =  − 0.10, 95% CI [ − 0.24,  − 0.002]). Respondents who lived in Taiwan reported lower levels of couple-level stigma and discrimination than those in Hong Kong; those who had lower levels of couple-level stigma experienced a lower degree of depression level. No significant indirect effect is established through parental acceptance (*B* =  − 0.003, 95% CI [ − 0.09, 0.09]). The direct effect between society of residence and depression levels was found to be nonsignificant in the presence of mediators and demographics (*B* = 0.33, *p* = .43). Hence, a full mediation model is found through couple-level stigma and discrimination on one’s depression level (see Fig. 1).

**Fig. 2:**
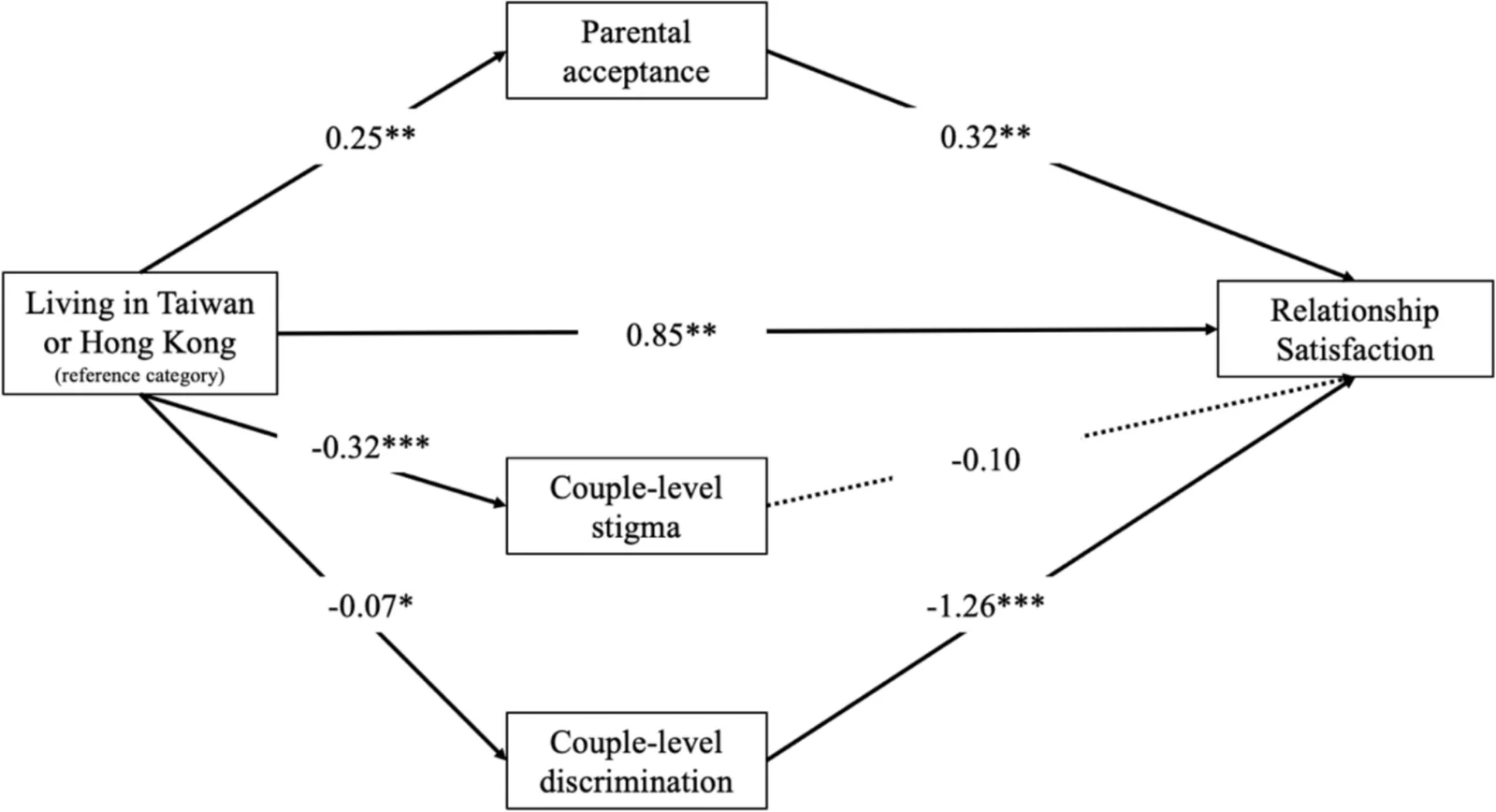
The mediating effect of parental acceptance and couple-level stigma and discrimination in the relationship between the societies one lived in and their relationship satisfaction level. Solid lines indicate significant paths, and dashed lines indicate nonsignificant paths. Demographic variables (gender, age, education, monthly income, and marital status) were controlled for in the analysis. *p < .05, **p < .01, ***p < .001

In Model 2, there are significant indirect effects between the societies they lived in and their relationship satisfaction through parental acceptance (*B* = 0.08, 95% CI [0.02, 0.17]) and couple-level discrimination (*B* = 0.09, 95% CI [0.004, 0.19]). Respondents who lived in Taiwan reported higher levels of parental acceptance and lower levels of couple-level discrimination, compared with those who lived in Hong Kong; higher levels of parental acceptance and lower levels of couple-level discrimination are associated with higher levels of relationship satisfaction experience. No significant indirect effect was found through couple-level stigma (*B* = 0.03, 95% CI [ − 0.07, 0.15]). The direct effect between the societies one lived in and their relationship satisfaction was significant (*B* = 0.85, *p* = .002) in the presence of mediators and demographics. Hence, both parental acceptance and couple-level discrimination partially mediated the relationship between the societies they lived in and their relationship satisfaction (see Fig. 2).

## Discussion

This study examined the difference in parental acceptance, couple-level minority stress, and individual and relational well-being among LGBQ+ individuals in committed same-sex relationships who lived in Taiwan, where same-sex couple relationships were legally recognized, and Hong Kong, where it is not. Respondents who lived in Taiwan reported higher parental acceptance, especially from their mothers, compared to those living in Hong Kong. Half of the respondents (50.0%) in Taiwan indicated that their mother accepted or strongly accepted their sexualities, while only around one-third (39.4%) of those in Hong Kong had an accepting mother. This may be partly explained by the legal recognition of same-sex couple relationships in Taiwan: Huang (2023) found that legal recognition contributes to the positive attitudinal changes among parents who have LGB children. Such legal recognition helps reduce parents’ worry about their children’s lives and have a sense of reassurance that their children’s same-sex relationship is legally protected by their government (Huang, 2023).

Our study revealed that nearly one-fourth of the respondents (22.7% in Taiwan, 22.6% in Hong Kong) met the criteria for probable clinical depression. Previous studies in Taiwan and Hong Kong indicated that only 11.1% and 14.6% of the general public met the criteria for probable depression, respectively (Mental Health Association of Hong Kong, 2017; National Health Research Institute & Health Promotion Administration, Ministry of Health and Welfare, 2017). Comparatively speaking, a much greater proportion of LGBQ+ individuals in same-sex relationships met the criteria for probable clinical depression, indicating a strong need for mental health intervention.

Notably, no significant difference in depression levels was found between respondents in Taiwan and Hong Kong. Thus, despite living in a society with legal recognition of same-sex couple relationships, LGBQ+ individuals in committed same-sex relationships may still face a broad range of stigma, discrimination, and rejection, which eventually bring psychological distress to them. Poor individual well-being in Taiwan might be related to the anti-marriage-equality campaigns in recent years and the referendum on same-sex marriage in 2018, where more than 7.7 million citizens in Taiwan voted against it (72% vs 28%) (BBC, 2018). The well-being of LGBQ+ individuals in Taiwan was significantly affected by the negative campaign messages and hostility expressed during the public debates on same-sex marriage (Chen et al., 2021; Lin et al., 2019; Wang et al., 2022). A study of over 1,000 LGBT Taiwanese found that 12.6% of respondents attempted suicide during the period of the referendum (Wang et al., 2022), a figure much higher than before the referendum (Wang et al., 2022). Suicidal ideation among non-heterosexual individuals was associated with perceived unfavorable attitudes toward same-sex marriage from their family members and heterosexual friends during the referendum (Ko et al., 2020). These results are consistent with previous studies in other countries. A longitudinal study in the United Kingdom revealed that exposure to negative campaign messages against same-sex marriage is associated with the negative psychological well-being of same-sex couples (Frost & Fingerhut, 2016). While same-sex couple relationships were legally recognized in Taiwan in 2019, time is needed for both the social climate toward LGBQ+ acceptance and the well-being of LGBQ+ individuals to evolve. Given that our data were collected less than two years after this legislative shift, we acknowledge that these findings likely capture the proximal, short-term effects of the policy change rather than a fully realized societal transformation—if there was one. Consequently, this study serves as a critical baseline for examining the initial stages of minority stress in the “post-marriage equality era” (Hull, 2018) by showing how social stigma and discrimination persist after having marriage equality and its impacts on LGBQ+ individuals’ well-being (Frost et al., 2022; Meyer, 2016).

We found evidence supporting the argument that couple-level stigma and discrimination are significant contributors to the variance in depression, after controlling for demographics, the societies they live in (with or without such policy), and their parental acceptance. Consistent with earlier studies (LeBlanc & Frost, 2020), respondents in Taiwan reported lower levels of couple-level stigma and discrimination than the Hong Kong respondents; lower levels of couple-level stigma and discrimination are associated with lower levels of depression. A full mediation model established that couple-level stigma and discrimination significantly mediate the relationship between the societies they live in and their depression levels. It is plausible that living in a society with legal recognition makes LGBQ+ individuals in committed same-sex relationships feel less stigmatized because their relationships are recognized by the government. In addition, they may experience less discrimination on the grounds of their same-sex relationship. These factors may lead to more favorable individual well-being outcomes. This empirical evidence helps contribute to the emerging literature on couple-level minority stress (LeBlanc & Frost, 2020; Neilands et al., 2020).

In terms of relational well-being, our study found that respondents in Taiwan reported a higher satisfaction in their same-sex relationship than their counterparts in Hong Kong: 75.8% felt satisfied with their relationship, compared to 65.8% in Hong Kong. Even after accounting for the mediators (parental acceptance, couple-level stigma, and discrimination) and demographic variables, the direct effect of regional location on relationship satisfaction remained significant. The finding is consistent with previous studies in North American and European countries. Same-sex couples who live in societies with same-sex marriage reported having a more satisfied relationship (Akers et al., 2023; Horne et al., 2022; MacIntosh et al., 2010). It may be explained by the suggestion that getting legal recognition for their same-sex relationships helps them feel that their relationships are being respected, making them feel more secure, which contributes to a more satisfying relationship (Lannutti, 2011; Riggle & Rostosky, 2007).

More importantly, our results showed that parental acceptance significantly contributes to the variance explained in relationship satisfaction, beyond the contribution of living in societies with legal recognition of same-sex couple relationships. It reveals how important parental acceptance is for LGBQ+ individuals in Chinese societies, even when they are adults and have established their own relationships. Their parents’ attitude toward their sexualities matters in their relationship quality, beyond the impacts of legal recognition of same-sex relationships. This can be attributed to the emphasis on family/kinship ties and harmony in Chinese societies, which makes LGBQ+ individuals value parental acceptance in their intimate relationships, as suggested in the “coming home” model (Chan, 2017; Chow, 2001). As such, this study contributed to the literature on the “coming home” model by revealing that getting parental acceptance is not merely a wish of LGBQ+ individuals in Chinese societies; parental acceptance, serving as a mediating role, can have actual impacts on their relational well-being, beyond the influences of legal recognition of same-sex relationships in the society they live in.

### Implications

Our findings showed that couple-level stigma and discrimination contribute to the individual and relational well-being of LGBQ+ individuals in committed same-sex relationships. Mental health interventions should attend to those specific stressors encountered by LGBQ+ individuals based on their same-sex relationships, in addition to those related to their sexual identity. Social and mental health service providers need to understand the unique experience LGBQ+ individuals face as same-sex couples and their interaction with their families, colleagues, friends, and community members, in order to provide better support for LGBQ+ individuals in same-sex relationships.

Also, as shown in our findings, parental acceptance plays a vital role in one’s individual and relational well-being. More emphasis and support should be provided for LGBQ+ individuals to handle their relationships with their parents. Social services should also be provided to parents who have LGBQ+ adult children to help them understand more about their children’s sexual orientation (Chan et al., 2022). Parents should be encouraged to maintain open communication with their children, in order to foster a supportive and caring family environment for both parents and their LGBQ+ adult children.

Moreover, policymakers should introduce legal recognition of same-sex couple relationships, which is crucial in reducing LGBQ+ individuals’ exposure to discrimination on the grounds of their sexual identity and same-sex relationships and in protecting their individual and relational well-being.

### Limitations

While the current study provides an important understanding of individual and relational well-being of LGBQ+ individuals in Chinese societies, the interpretation of the findings needs to be made with caution. First, even though legal recognition of same-sex couple relationships is the major difference in LGBQ+ rights between Taiwan and Hong Kong, other political, social, and cultural factors may also contribute to the difference in terms of parental acceptance, couple-level minority stress, and LGBQ+ individuals’ well-being in these two societies. Qualitative research is needed to investigate the reasons behind the difference observed in this study, with a particular focus on whether, and how, such legal recognition affects LGBQ+ individuals’ lived experience, their parental acceptance, and their relationships with their same-sex partners (Kong, 2024; Wong, 2025b).

Second, the study adopts a non-probability sampling framework due to the absence of a sampling framework for LGBQ+ individuals in Hong Kong and Taiwan (Lau et al., 2023; Suen & Chan, 2021). The sample was disproportionately younger and more educated and had lower monthly income than the average citizen. Thus, the findings might not be generalizable to the entire LGBQ+ population in Taiwan and Hong Kong. Third, because this study is comparative and cross-sectional in nature, a causal relationship cannot be established.

Third, a notable limitation of this study is its reliance on a single-item measure of parental acceptance, which lacks the psychometric depth of validated, multi-item scales capable of assessing support across diverse sources (parents vs. peers) and dimensions (identity vs. relationship) (Holmberg & Blair, 2016). In particular, our measure captures parental acceptance of sexual orientation (identity) rather than support for the specific same-sex relationship. Although conceptually distinct, these dimensions are often functionally inseparable for same-sex couples due to “attributional ambiguity,” in which a lack of relationship support may be interpreted as a reflection of identity-based prejudice (Blair & Holmberg, 2019). Furthermore, the Chinese “coming home” strategy, in which partners are often introduced as “friends” to maintain familial harmony, makes relationship-specific support difficult to measure empirically, as the romantic nature of the union may remain undisclosed to the family of origin (Huang & Brouwer, 2018). Future research should use multidimensional scales to disentangle how legal recognition differentially shapes identity-based acceptance versus relationship-level support as social visibility evolves in Chinese societies.

Moreover, this study relies on self-reported measures of depressive symptoms, relationship satisfaction, and couple-level minority stress. Parental acceptance was reported by the respondents based on their subjective perceptions. Further studies should include clinical diagnosis, if possible, in the analysis (Cuijpers et al., 2010). Also, future research could consider recruiting parents with LGBQ+ children, in order to understand the dyadic interaction between parents and children (Brainer, 2017; Chan, 2022; Chan et al., 2022; Huang, 2023).

Furthermore, this study only included cisgender individuals who were in committed relationships because the study focuses on the legal recognition of same-sex couple relationships. Transgender and gender non-binary individuals’ union usually involves other laws and policies, such as legal gender recognition (Dunne, 2014). Transgender and gender non-binary individuals may face unique stressors and challenges in their families, and under the current law, policy, and social climates in their societies. Further studies should focus on transgender and gender non-binary individuals and study the impacts of legal recognition of same-sex couple relationships and parental acceptance on their well-being.

Finally, this study was conducted in 2021–2022, less than two years after legal recognition of same-sex couple relationships in Taiwan. The findings may have only captured the short-term impacts of the policy change. It is acknowledged that deeply rooted social constructs, such as parental acceptance and structural stigma, likely require a more extended trajectory for substantive transformation (Frost et al., 2022). Nonetheless, our findings capture the proximal impacts of this legislative shift, providing a critical baseline for future research (Wong, 2025a). A longitudinal follow-up study is needed to understand the long-term impacts of the policy on same-sex couples’ well-being, their perceived parental acceptance, and the stigma and discrimination they experience in their societies.

## Conclusion

This study examined the difference in well-being among LGBQ+ individuals in committed same-sex relationships who live in Taiwan (a society with legal recognition of same-sex couple relationships) and Hong Kong (without the policy). It was found that LGBQ+ individuals in Taiwan have better relational well-being than their counterparts in Hong Kong, but there is no significant difference in their individual well-being. This indicates that even after legal recognition, LGBQ+ individuals may still face a wide range of stigma and discrimination, which eventually affects their well-being. Also, LGBQ+ individuals who have parental acceptance tend to have a more satisfied relationship with their same-sex partners, beyond the impacts of having legal recognition on their same-sex relationships in the societies they live in. Mental health support is needed for LGBQ+ individuals in same-sex relationships in Chinese societies, with a particular focus on unique stressors experienced by same-sex couples and their relationships with their parents. This study contributed to the emerging literature on couple-level minority stress, the debates on minority stress in the “post-marriage equality era,” the importance of parental acceptance, and the “coming home” model in Chinese sexualities studies.

## Data Availability

The datasets generated and/or analyzed during this study are not publicly available due to concerns regarding confidentiality and data protection.
